# Assessing the Contribution of the Oscillatory Potentials to the Genesis of the Photopic ERG with the Discrete Wavelet Transform

**DOI:** 10.1155/2016/2790194

**Published:** 2016-12-22

**Authors:** Mathieu Gauvin, Allison L. Dorfman, Nataly Trang, Mercedes Gauthier, John M. Little, Jean-Marc Lina, Pierre Lachapelle

**Affiliations:** ^1^Department of Ophthalmology, Research Institute of the McGill University Health Centre/Montreal Children's Hospital, Montréal, QC, Canada; ^2^Department of Neurology-Neurosurgery, Research Institute of the McGill University Health Centre/Montreal Children's Hospital, Montréal, QC, Canada; ^3^Département de Génie Électrique, École de Technologie Supérieure, Montréal, QC, Canada; ^4^Centre de Recherches Mathématiques, Montréal, QC, Canada

## Abstract

The electroretinogram (ERG) is composed of slow (i.e., a-, b-waves) and fast (i.e., oscillatory potentials: OPs) components. OPs have been shown to be preferably affected in some diseases (such as diabetic retinopathy), while the a- and b-waves remain relatively intact. The purpose of this study was to determine the contribution of OPs to the building of the ERG and to examine whether a signal mostly composed of OPs could also exist. DWT analyses were performed on photopic ERGs (flash intensities: −2.23 to 2.64 log cd·s·m^−2^ in 21 steps) obtained from normal subjects (*n* = 40) and patients (*n* = 21) affected with a retinopathy. In controls, the %OP value (i.e., OPs energy/ERG energy) is stimulus- and amplitude-independent (range: 56.6–61.6%; CV = 6.3%). In contrast, the %OPs measured from the ERGs of our patients varied significantly more (range: 35.4%–89.2%; *p* < 0.05) depending on the pathology, some presenting with ERGs that are almost solely composed of OPs. In conclusion, patients may present with a wide range of %OP values. Findings herein also support the hypothesis that, in certain conditions, the photopic ERG can be mostly composed of high-frequency components.

## 1. Introduction

The electroretinogram (ERG) waveform is characterized with a negative a-wave that is generated by the photoreceptors followed by a larger positive b-wave, which originates from the bipolar and Müller cells [1]. Low-voltage high-frequency oscillations, known as the oscillatory potentials (OPs), are also often seen riding on the ascending limb of the b-wave [2, 3]. Although their origin remains debated, it has been suggested that they would represent inner retinal potentials generated by neuronal interactions that might involve bipolar, amacrine, and/or ganglion cells [3–6]. Of interest, while previous studies have shown that, in some retinopathies (such as in diabetic retinopathy or central retinal vein occlusion), the OPs appeared to be selectively more affected compared to the relatively better preserved a- and b-waves [7–9], to date no study has reported ERG responses which seemed to be solely composed of OPs or where the OPs appeared to be selectively better preserved than the slower components of the ERG (i.e., a- and b-waves).

Notwithstanding the above, it must be remembered that traditionally the OPs are extracted using a bandpass filtering technique in order to remove the low-frequency components (i.e., a- and b-waves) from the broadband ERG signal. Unfortunately, bandpass filtering can generate signal distortion such as phase lag, ringing artefacts, and/or attenuation of OP amplitude which can lead to erroneous measures and even, in some instances, “create” artifactual OPs [10, 11]. As a remedy to the latter, it was suggested to quantify the OPs in the frequency domain with the use of the fast Fourier transform (FFT) [6, 12]. Unfortunately, given that the FFT quantifies the power level of all the frequency components contained within a signal (such as the ERG), whether they are time-locked to the stimulus or not (i.e., no temporal resolution), its use can lead to erroneous interpretations [10, 13–15]. Fortunately, the latter limitation can be easily overcome with the use of the discrete wavelet transform (DWT), which is somewhat of an improved FFT since it includes both temporal and frequency resolutions [10, 13–18].

With the above in mind, we sought to determine the contribution of OPs to the building of the photopic ERG waveform obtained from normal subjects and patients and to examine whether a signal mostly composed of OPs could also exist.

## 2. Methods

### 2.1. Selection of ERG Responses Analysed

Analysis was performed on photopic ERGs (bandwidth: 1–300 Hz; flash intensities: −2.23 to 2.64 log cd·sec·m^−2^ in 21 steps of ~0.2 log-unit; background: 30 cd·m^−2^; averages of 10 to 300 flashes per response) obtained from 40 normal subjects. Results were compared to photopic ERG responses obtained from patients (bandwidth: 1–300 Hz; flash intensities: 0.64 log cd·sec·m^−2^; background: 30 cd·m^−2^; averages of 10 to 300 flashes per response) affected with retinopathies known to selectively abolish the OPs [i.e., diabetic retinopathy (DR) and central retinal vein occlusion (CRVO)] [7–9]. These patients were selected on the basis of the clinical findings that were characteristic of the disease condition (i.e., mostly fundus appearance and in the case of CRVO, unilateral presentation). Results were also compared to photopic ERG response obtained from patients (bandwidth: 1–300 Hz; flash intensities: 0.64 log cd·sec·m^−2^; background: 30 cd·m^−2^; averages of 10 to 300 flashes per response) affected with an advanced retinal degeneration (mostly* retinitis pigmentosa*) and where, on visual inspection (by a naïve observer: CS), fast oscillations, time-locked to the stimulus and in the frequency range of the OPs (as estimated using a template of a normal OP response) appeared to be the most prominent features of the response. An informed consent form was signed by each subject and the protocol was previously approved by the Institutional Review Board and was conducted in accordance with the declaration of Helsinki. Additional details regarding the ERG setup and recording procedures were previously published by us [19–23].

### 2.2. Analysis of ERG Responses

**Figure 1:**
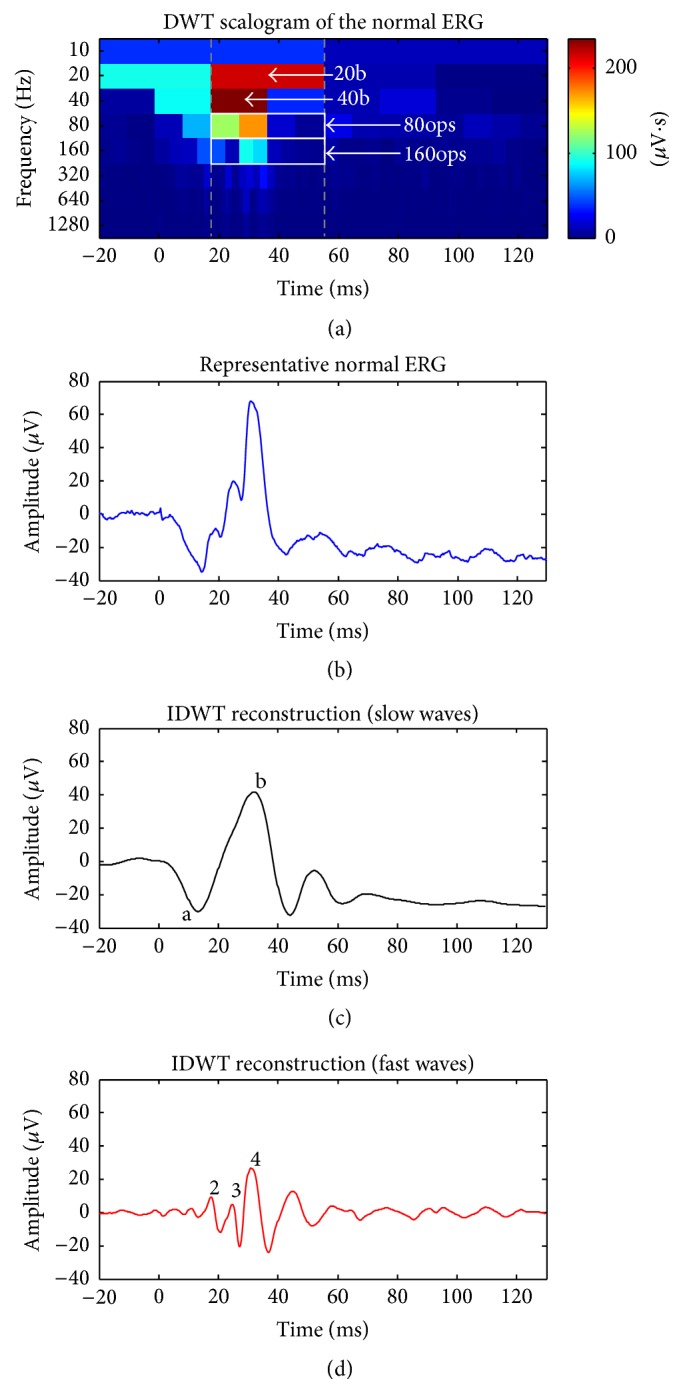
Computation of the percentage of oscillatory potentials (%OPs) contributing to the ERG response. The DWT scalogram (a) is shown above the broadband ERG (b). Temporal sections of the scalogram that were taken into consideration when calculating the %OPs are outlined by the dotted gray lines in order to ease appreciation of the time-frequency region in question. The DWT descriptors (20b, 40b, 80ops, and 160ops) that were used to compute the %OPs are identified on the scalogram. The inverse DWT (IDWT) reconstructions of the slow (a- and b-waves, indicated by the corresponding letters) and fast (OPs, indicated by corresponding numbers) waves are shown as the black and red traces (panels (c) and (d)), respectively.

The DWT of each ERG signal was computed using the fast wavelet transform algorithm of Mallat [35] implemented with Matlab R2015a. The DWT generates scalograms (Figure 1(a)) which display the energy (*z*-axis) of the signal (maximal values are shown in red; lowest values in blue) as a function of time (*x*-axis) and frequency (*y*-axis). As previously demonstrated by us and others [10, 13, 15–18], this time-frequency approach allows for the identification of energy descriptors, each defined with their respective time and frequency coordinates. The DWT yields measurements of the photopic b-wave (found in the 20 and 40 Hz bands) and OPs (found in the 80 and 160 Hz bands) through the quantification of their respective associated wavelet coefficients [10, 16, 17]. As exemplified in the scalogram of Figure 1(a), two DWT descriptors were used to quantify the 20 Hz and 40 Hz b-wave energy (identified as 20b and 40b) and another two were used to quantify the 80 Hz and 160 Hz OPs energy (identified as 80ops and 160ops), each computed by summating values outlined by the white boxes, respectively. Similar to bandpass filtering, the inverse DWT (IDWT; see [10, 17]) can be used to specifically reconstruct the low-frequency (20 and 40 Hz) and high-frequency (80 and 160 Hz) bands of the signals, which are specific to the slow and fast waves of the ERG. As shown in Figures 1(c) and 1(d), the IDWT confirms that the 20 and 40 Hz descriptors quantify the slow waves (a- and b-waves, as identified in Figure 1(c)) of the ERG, while the 80 Hz and 160 Hz descriptors quantify the fast waves (OPs, identified as 2, 3, and 4 in Figure 1(d)). Consequently, these DWT descriptors were used to quantify the percent contribution of the OPs (%OPs) to the ERG according to the following equation:

(1) %OPsOPs  EnergyBroadband  ERG  Energy=80ops+160ops20b+40b+80ops+160ops.

### 2.3. Statistical Analysis

Descriptive statistics (mean and standard deviation [SD]) of the %OPs were obtained from our selected patients and from our normal subject cohort at each of the 21 stimulus intensities. The coefficient of variation (CV) of selected groups was computed as the SD divided by the mean and multiplied by 100. To further confirm that each of our selected pathological ERGs had a significantly (*p* < 0.05) lower (DR and CRVO patients) or higher (end-stage retinal degeneration patients) than normal %OPs, individual one-way *Z*-tests were used with a critical value set at 1.645 [36]. Thus *Z*-scores of ±1.645 will indicate a significantly higher/lower (*p* < 0.05) than normal contribution of the OPs to the broadband ERG, respectively. Finally, an unpaired *t*-test (one-tail) was also used to validate whether our group of advanced retinal degeneration patients had a significantly (*p* < 0.05) higher percentage of OPs (%OPs) contributing to their ERGs.

## 3. Results

### 3.1. %OPs Contribution to the Normal ERG Response

**Figure 2:**
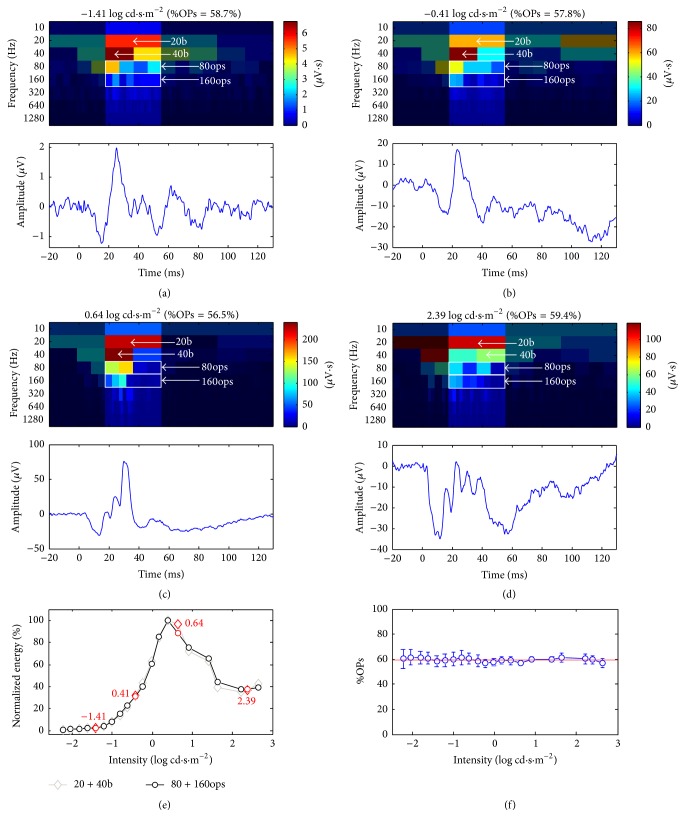
Normal ERGs (obtained at four different stimulus intensities) which show a relatively constant %OPs contribution (from (a) to (d): 58.7%; 57.8%; 56.5%; 59.4%). The four examples are shown in increasing order of stimulus intensities from panels (a) to (d). The DWT scalograms are shown at the top of each broadband ERG. Sections of the scalogram that were not considered in the %OPs computation were shaded to facilitate comparison. The descriptors (20b, 40b, 80ops, and 160ops) that were used to compute the %OPs are identified on each scalogram. (e) Normalized luminance-response functions of the DWT descriptors of the b-wave (20b + 40b) and OPs (80ops + 160ops). The red markers refer to the intensities shown from panels (a) to (d). (f) Mean ± SD %OPs values obtained at each stimulus intensity.

Representative ERG waveforms of the photopic luminance-response (LR) function obtained at four different stimulus intensities are shown in Figures 2(a) to 2(d) and the positions of these responses on the photopic LR function are indicated with a red diamond in Figure 2(e). As shown in Figure 2(a), no OPs could be detected on the rising phase of the b-wave evoked to the dimmest stimulus (i.e., −1.41 log cd·m^−2^) as well as in the response (Figure 2(b)) evoked to a stimulus one log-unit brighter (−0.41 log cd·m^−2^), despite a 10-fold increase in b-wave amplitude (i.e., from ~3 to ~30 *μ*V). The latter contrasts with the ERG evoked to the 0.64 log cd·s·m^−2^ stimulus (Figure 2(c)) where two prominent OPs are seen on the ascending phase of the b-wave. A further increase in stimulus intensity to 2.39 log cd·s·m^−2^ (Figure 2(d)) will yield a b-wave of reduced amplitude (approximately 30 *μ*V) compared to the 0.64 log cd·s·m^−2^ response but with more OPs. The above suggests, at least from visual inspection, that the prominence of OPs increases with stimulus intensity, but not necessarily with b-wave amplitude. However, as indicated at the top of the four representative scalograms (Figures 2(a)–2(d)), when we measure the %OPs content of these ERGs, nearly identical values are found (%OPs = 58.7; 57.8; 56.5; and 59.4 for intensities of −1.41; −0.41; 0.64; and 2.39 log cd·s·m^−2^, resp.). This is best exemplified with the graphs of Figures 2(e) and 2(f) reporting the luminance-response functions of DWT descriptors of the b-wave (i.e., 20b + 40b; gray trace; diamond markers) and OPs (i.e., 80ops + 160ops; black traced; round markers) are nearly identical (once normalized). Therefore, as reported in Table 1 and illustrated in Figure 2(f), in normal subjects, across all of the 21 stimulus intensities (close to 5 log-unit range), the %OPs varied between 56.6 (smallest value) and 61.6 (highest value) and was thus significantly less variable than the b-wave amplitude which varied between 1.2 *μ*V (lowest value) and 115.52 *μ*V (highest value). The above demonstrate not only that the %OPs is nearly stimulus-independent but also that it varies very little (see Table 1) as the mean %OPs coefficient of variation (CV) of all the intensities considered was of 6.34% compared to 87.37% for the b-wave amplitude.

### 3.2. Pathological ERG Responses Presenting with a Reduced %OPs Contribution

**Figure 3:**
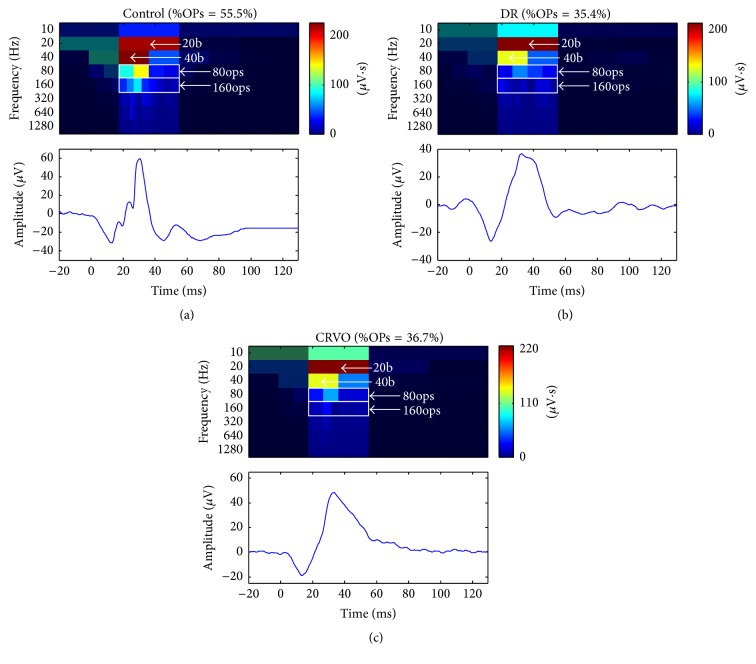
(a) %OP contribution to the photopic ERG response obtained from a normal subject and patients with diabetic retinopathy (DR) (b) and CRVO (c). All photopic ERG responses were evoked to the 0.64 log cd·s·m^−2^ stimulus. Sections of the scalogram that were not considered in the %OPs computation were shaded to facilitate comparison. The descriptors (20b, 40b, 80ops, and 160ops) that were used to compute the %OPs are localized on each scalogram. The %OPs is shown at the top of each DWT scalogram.

As predicted from previous studies [7–9], ERGs recorded from patients affected with diabetic retinopathy (DR, Figure 3(b)) or central retinal vein occlusion (CRVO, Figure 3(c)) presented no evidence of OPs on the rising phase of the b-wave. These conditions allowed us to investigate if our method of determining the OP content with the DWT (i.e., the %OPs) would be reduced in situations where the OPs are selectively attenuated. The latter is clearly reflected with the significantly reduced %OPs of 35.4% (*Z*-score: −8.48; *p* < 0.05) and 36.7% (*Z*-score: −7.96; *p* < 0.05) that we found for DR and CRVO, respectively.

### 3.3. Pathological ERG Responses Presenting with a Higher %OPs Contribution

**Figure 4:**
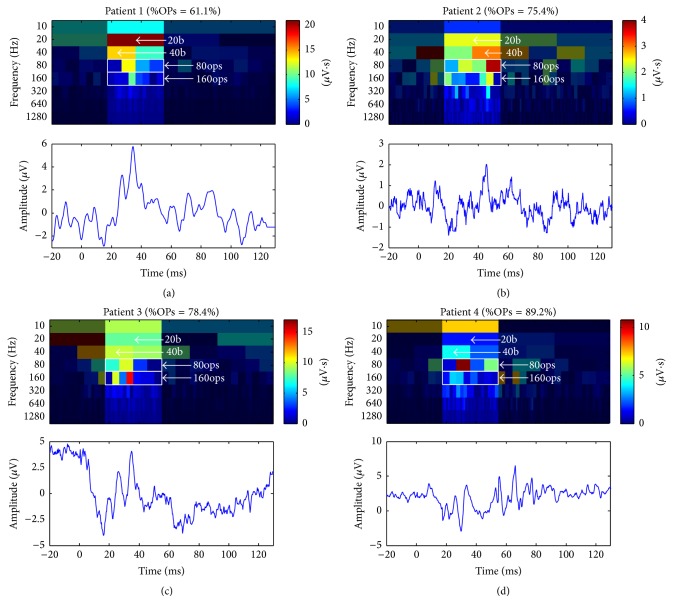
Pathological ERGs (obtained from four different end-stage RP patients) which show a significantly higher %OPs contribution. The four cases are shown in increasing order of %OPs from panels (a) to (d). In each case, the DWT scalogram is shown on the top of the broadband ERGs. Temporal sections of the scalogram that were not considered in the %OPs computation were shaded to ease appreciation for the region in question. The descriptors (20b, 40b, 80ops, and 160ops) that were used to compute the %OPs are shown on each scalogram. The %OPs is shown at the top of each DWT scalogram.

Analysis of ERGs obtained from our databank revealed that the patients in this group were mostly affected with retinitis pigmentosa (RP), accounting for a total of 15 cases. The clinical findings, such as visual fields, visual acuities, and rod ERG amplitudes, which are reported in Table 2, are indicative of end-stage retinopathies. Using the above-defined DWT technique, we determined that the overall percentage of OPs contribution was significantly greater than that obtained from control (%OPs of 74.1% ± 8.1% versus 56.6% ± 2.5%, resp., *p* < 0.05) using the same suprathreshold photopic ERG waveform (0.64 log cd·s·m^−2^). Representative examples of enhanced %OPs ERGs are shown in Figure 4 and data from patients are reported in Table 2. For example, as shown in Figure 4(a), patient 1 (also referred to as patient 1 in Table 2) presented with a %OPs of 61.1%, a value that is slightly but nonetheless significantly higher than control (*Z*-score: 1.78; *p* < 0.05). In contrast, the ERGs of patients 2 and 3 (Figures 4(b) and 4(c)) presented with a more pronounced enhancement of the %OPs parameter to 75.4% and 78.4%, respectively (*Z*-scores: 7.46 and 8.67; *p* < 0.0001). Finally, the ERG tracing and scalogram obtained from patient 4 (Figure 4(d)) disclosed a waveform that is almost solely composed of OPs with a %OPs of 89.2% (*Z*-score: 13.14; *p* < 0.0001), the highest %OPs value obtained from our patient cohort.

### 3.4. Example of %OPs Progression with Disease Process

**Figure 5:**
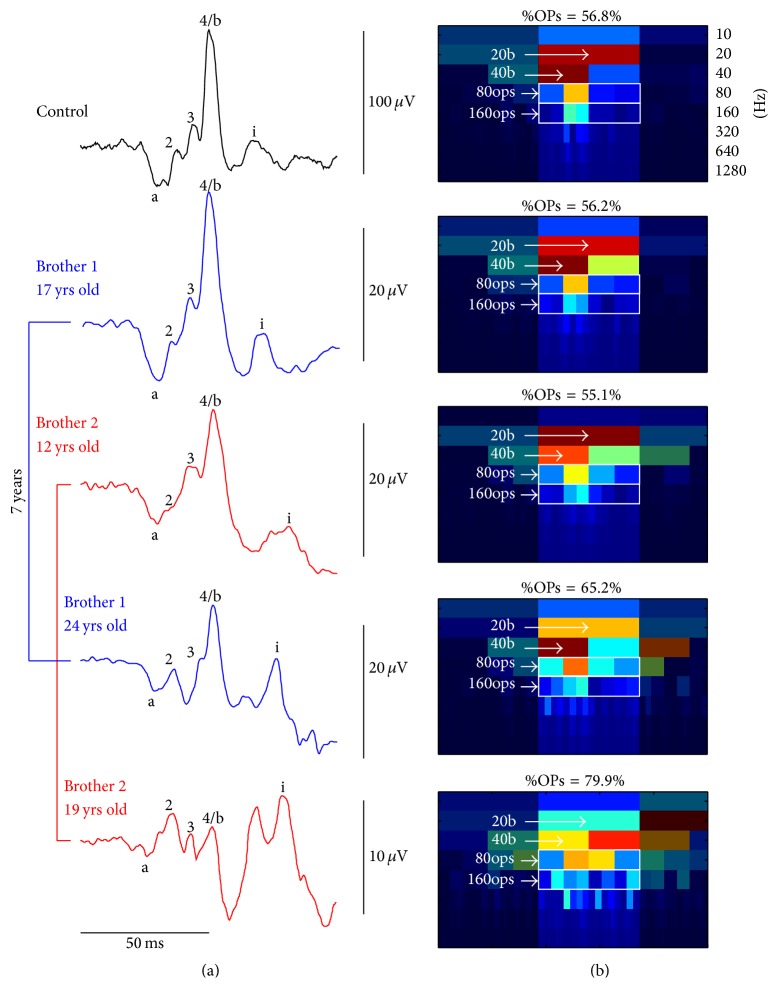
(a) A control ERG (black tracing) is shown with the ERG follow-up of patients #16 (blue tracings) and 17 (red tracings), at first visit (12 and 17 years of age, resp.) and at second visit (24 and 19 years of age, resp.). The a-, b-, and i-waves are indicated by their corresponding letters and OP2, OP3, and OP4 by their corresponding numbers. Calibration:* horizontal:* 50 ms;* vertical:* as shown to the right of each tracing. (b) Corresponding DWT scalograms and associated %OPs. Descriptors (20b, 40b, 80ops, and 160ops) that were used to compute the %OPs are localized on each scalogram. Note that, in the scalogram corresponding to the 2nd visit of Brother 2 (bottom tracing), the 40b descriptor was identified using the value of the left rectangle (yellow) as the higher energy red rectangle was time-locked to the i-wave.

Figure 5(a) shows the photopic ERGs obtained from two brothers (aged 12 and 17 years at initial exam and identified as patients 16 and 17, resp., in Table 2) affected with choroideremia. On initial examination, both brothers presented with severely attenuated ERGs, albeit with normal looking morphologies, that contrast with the more oscillatory ERG waveforms that we obtained seven years later. The corresponding DWT scalograms indicate that while, at the initial visit, the %OPs was within the normal limits (i.e., 56.2% and 55.1%), seven years later it had increased to 65.2% and 79.9%, suggesting that disease progression was most detrimental to the slow frequency generators of the ERG (e.g., b-wave).

## 4. Discussion

The purpose of this study was to determine the energy contribution of the oscillatory potentials to the building of the photopic ERG response and to investigate if an ERG could be mostly composed of OPs. Our study demonstrated that, in normal ERGs, the %OP value is relatively constant (overall CV = 6.3%), not stimulus-dependent (range 56.6% to 61.6%), and consequently not influenced by the absolute amplitude of the ERG b-wave (which varied between 1.2 and 115.4 *μ*V; overall CV = 87.4%). This nearly stimulus-independent %OPs value can be explained by the fact that the b-wave and OPs covary across the luminance-response function of the normal photopic hill, increasing in the initial portion, reaching a plateau, and then decreasing with brighter intensities. The latter is supported by previous studies which showed that OPs and b-wave parameters as well as their luminance-response (LR) functions were highly covariant [17, 37–41]. For example, a correlation coefficient of 0.78 (*p* < 0.05) was previously reported between the b-wave amplitude and the sum of OPs (SOP) amplitude [39] and an even higher correlation coefficient of 0.98 (*p* < 0.05) was previously found between LR functions of the b-wave and OPs energy [17]. However, the nearly stimulus-independent %OPs value can be surprising to some given that, on visual inspection, waveforms evoked to progressively brighter stimuli appear to be gradually more oscillatory as exemplified in Figure 2. Nevertheless, DWT analysis suggests that more prominent and more numerous OPs on the ascending limb of the b-wave do not necessarily indicate a larger contribution of the OPs to the building of the ERG since the %OPs value was shown to be nearly stimulus-independent.

The above range of normal %OPs (56.6% to 61.6%) also demonstrates that, in normal subjects, the summed OPs energy contributes to more than half (%OPs > 50%) of the total energy contained within the ERG waveform. Based on normal data included in previously published studies where OPs were extracted using bandpass filtering, we derived (see computation details in Table 3 caption) time domain %OPs (TD%OPs) values (reported in Table 3) as low as 26% [24] or as high as 112% [31]. As aforementioned, bandpass filtering can generate signal distortions such as phase lag, ringing artefacts, attenuation of OPs amplitude, and/or artefactual enhancement of OPs. The latter most probably accounts for the very large variation of the time domain (TD) derivation of the %OPs reported in Table 3. Intuitively, the “real” %OPs value should lie somewhere between the two extremes (i.e., close to the mean of all values). Of interest, the average normal TD%OPs derived from all previous studies (Table 3) was of 57.50% ± 30.01%, a value that is not significantly different (*p* > 0.05) from the %OPs of the normal photopic ERG waveform (irrespective of stimulus intensity) that we quantified with the DWT (59.10% ± 3.75% as per Table 1). The latter would suggest that the %OPs computed with the DWT represents an accurate and highly reproducible (CV as low as 3.26% for the 0.9 log cd·s·m^−2^ in Table 1) method to estimate the %OPs contribution to the building of the ERG.

**Figure 6:**
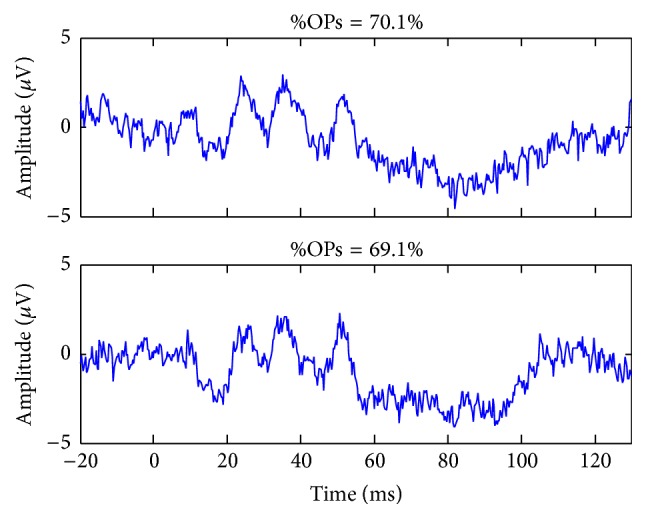
Representative test-retest repeatability of the %OPs. Tracings (top and bottom panels) were recorded twice in the same RP patient. The %OPs is shown at the top of each tracing.

Contrasting with the above findings obtained from normal subjects, data obtained from our patients did not present a constant %OPs value and showed a wide range of %OPs, some presenting with a relatively suppressed %OPs (such as 35.4%, CRVO ERG) and others with a relatively enhanced %OPs (such as 89.2%, end-stage RP ERG). Our analysis revealed that pathological ERGs presenting with OPs more prominent than normal (on visual inspection) disclosed a higher than normal %OPs, with some almost solely composed of OPs. However, ERGs from RP patients (see Figure 4) were sometimes of very low amplitude (and some with poor signal-to-noise ratio (SNR), e.g., Figure 4(b)) and, as such, one wonders if the %OPs value was repeatable within subjects. To investigate the latter, a representative patient was recorded twice to test for reproducibility (Figure 6). Despite the very low SNRs of 1.3 and 1.6 obtained on test and retest, respectively, the response morphology was found to be reproducible and so was the resulting %OPs (test: %OPs = 70.1%; retest: %OPs = 69.1%). The latter suggest that a repeatable measurement of the %OPs can be obtained, even in low-SNR ERGs. To our knowledge, the findings reported herein demonstrate, for the first time, that in certain conditions the photopic ERG can be mostly (or solely) composed of high-frequency components, known as the OPs.

Interestingly, studies reporting on (slow sequence) multifocal ERGs of normal macaque monkeys revealed that responses obtained from the central retina were more oscillatory in nature and characterized with highly prominent high-frequency waves [6]. Similarly, focal macular ERG responses evoked from patients affected with RP also presented OPs that were relatively better preserved compared to the a- and b-waves, thus suggesting that macular responses are more oscillatory in nature [42], a finding also confirmed with focal macular ERGs obtained in a rabbit model of RP [43]. Given that most of our patients (as summarized in Table 2) that presented with highly prominent OPs had a very constricted visual field (which is a common finding in end stage of rod/cone dystrophies such as RP), the more oscillatory ERG responses that we found could find an explanation in the relatively better preserved central (macular) function. The above would therefore suggest that the macular region would contain a greater proportion of inner retinal cells which are suggested to be involved in OPs generation (i.e., amacrine, bipolar, horizontal, or ganglion cells) compared to retinal cells involved in the genesis of the b-wave (i.e., bipolar and Müller cells). This claim would find support from previously published findings on retinal cell distribution in nonhuman primates [32–34]. Based on these studies, we computed (Table 4) a significantly greater bipolar-to-Müller cell ratio (BMR) at the fovea compared to more peripheral regions (i.e., 24, 40, and 65 degrees from fovea), thus supporting our claim.

In conclusion, normal subjects disclosed a relatively constant and highly reproducible %OPs values while patients presented with a significantly larger range of %OPs values. We postulated that, in some circumstances, a pathological ERG signal could be mostly (or solely) composed of OPs. Our analyses did indeed reveal that some ERGs were almost solely composed of OPs. Findings herein therefore support the hypothesis that, in certain conditions, the photopic ERG can be mostly composed of high-frequency components, known as the OPs. Furthermore, the significantly wider range of %OPs values measured in patients (with variable b-wave amplitudes) would suggest that one of the two ERG components (b-wave or OPs) might be relatively more affected than the other and the DWT therefore represents a worthwhile approach to help in the segregation of pathological ERGs.

## Figures and Tables

**Table 1:** Normal variation of the %OPs as a function of the 21 stimulus intensities. Column 1: stimulus intensities. Column 2: mean %OPs values obtained at each stimulus intensity. Column 3: coefficient of variation (CV) of the %OPs at each stimulus intensity. Column 4: mean b-wave amplitude obtained at each stimulus intensity. Column 5: CV of the b-wave amplitude at each stimulus intensity. Range, mean (±1 SD).

Intensity (log cd·s·m^−2^)	Mean %OPs (%)	%OPs CV (%)	b-wave amplitude (*μ*V)	b-wave amplitude CV (%)
−2.23	60.27	12.64	1.23	33.74
−2.04	61.56	9.96	1.54	30.73
−1.81	61.27	8.41	1.93	26.79
−1.62	60.75	7.56	2.67	20.10
−1.41	58.19	7.15	3.70	20.28
−1.21	59.23	8.35	5.02	18.27
−1.0	60.08	9.13	10.86	17.60
−0.8	61.41	9.40	18.24	27.41
−0.62	60.47	6.66	28.61	21.25
−0.41	58.67	7.56	39.02	21.39
−0.23	56.60	6.12	51.26	23.68
−0.02	57.76	5.13	76.81	25.69
0.17	59.02	4.56	102.48	22.73
0.39	58.86	5.51	115.42	22.04
0.64	56.64	4.42	112.78	17.25
0.90	59.96	3.26	100.49	18.36
1.40	59.89	3.68	89.71	20.87
1.63	61.38	5.98	57.92	18.55
2.15	60.19	6.61	47.64	20.92
2.39	59.56	5.23	46.35	17.96
2.64	56.99	6.35	49.35	24.36
Range	56.6–61.6	3.3–12.6	1.2–115.4	17.3–33.7
Mean ± SD	59.10 ± 3.75	6.82 ± 2.29	45.86 ± 40.10	22.48 ± 4.13
Total CV	6.34		87.37	

**Table 2:** Ophthalmological and electrophysiological findings of the end-stage patient cohort included in this study. Patient number, age, visual acuity, visual field findings, rod- and cone-mediated ERG responses, diagnosis, %OPs values, and associated *Z*-scores are each indicated in individual columns. The %OPs contributing to the cone ERG response and associated *Z*-scores are shown in the last two columns, respectively. The critical *z*-score was set to a value of 1.645 and therefore any value > 56.6 + 1.645*∗*2.5 (i.e., 60.7) is considered to be significant. LP: light perception; flat: extinguished ERG; RP: retinitis pigmentosa.

Patient	Age	Visual acuity	Visual field	Rod ERG	Cone ERG	Diagnosis	%OPs	*Z*-scores
1	62	20/50	<20°	Flat	<10%	RP	61.1	1.78
2	61	20/400	<10°	Flat	<5%	RP	75.4	7.46
3	51	20/200	*∗*	<2%	<10%	RP	78.4	8.67
4	9	20/20	<15°	Flat	<10%	RP	89.2	13.14
5	44	20/100	<10°	Flat	<10%	RP	82.0	10.30
6	35	LP	<10°	Flat	<2%	RP	86.5	11.92
7	75	20/400	<10°	Flat	<2%	RP	74.9	7.46
8	6	20/30	N/A	Flat	<5%	RP	73.4	6.65
9	35	20/20	N/A	Flat	<5%	RP	67.1	4.21
10	73	20/400	<10°	Flat	<5%	RP	78.7	9.08
11	39	20/20	<15°	<1%	<10%	RP	64.3	3.00
12	51	20/50	<15°	Flat	<10%	RP	68.6	5.02
13	44	20/20	†	Flat	<10%	RP	65.4	3.40
14	58	20/60	<10°	Flat	<5%	RP	78.5	8.67
15	53	20/100	<10°	Flat	<10%	RP	71.8	6.24
16	24	20/25	<20^°‡^	<5%	<15%	Choroideremia	65.2	3.40
17	19	20/30	<15^°‡^	<5%	<10%	Choroideremia	79.9	9.49

^*∗*^Presence of an annular scotoma

^†^Large temporal scotoma with isopter IVe and <10% with isopter IIe

^‡^Isopter IIe.

**Table 3:** Time domain (TD) derivation of the TD%OPs contribution to the photopic ERG from previous studies (column 1). Column 2: TD%OPs values are presented in increasing order and were computed as the sum of OPs (SOP) amplitude divided by the associated b-wave amplitude and multiplied by 100. Column 3: the type of filters that were used. N/A indicates unspecified filter type. Columns 4 and 5: ERG and OPs cutoff frequencies that were used for bandpass filtering. Column 6: the attenuation that was used at the specified cutoff frequencies. N/A indicates unspecified attenuation.

Papers	TD%OPs (%)	Filter type	ERG cutoff (Hz)	OPs cutoff (Hz)	Attenuation (dB)
Miyake, 1990 [24]	26	N/A	5.3–100	100–300	N/A
Lachapelle et al., 1993 [25]	28	N/A	1–1000	100–1000	6
Peachey et al., 1991 [26]	36	N/A	1–1000	100–1000	3
Kizawa et al., 2006 [7]	38	N/A	0.5–1000	100–1000	N/A
Lachapelle et al., 1998 [27]	44	N/A	1–1000	100–1000	6
Holopigian et al., 1992 [28]	48	N/A	1–300	100–1000	N/A
Rufiange et al., 2005 [22]	69	N/A	0.3–500	75–500	6
Fortune et al., 2004 [29]	75	Blackman filter	0.3–3000	70–280	3
Kergoat et al., 2001 [30]	99	N/A	1–1500	50–1500	N/A
Peachey et al., 1987 [31]	112	N/A	1–1000	100–1000	3

**Table 4:** Derivation of the bipolar-to-Müller cell ratio at different retinal eccentricities from previous nonprimate studies.

Eccentricity	Cones density (cells/mm^2^)^*∗*^	Cone bipolar cells density (cells/mm^2^)^†^	Müller cells density (cells/mm^2^)^‡^	Bipolar-to- Müller cell ratio
Fovea	220,000	244444	30,000	8.15
24 deg.	35,000	4730	25,000	0.19
40 deg.	22,000	4889	20,000	0.24
65 deg.	17,000	1809	15,000	0.12

^*∗*^Adapted from Wilder et al. [32].

^†^Adapted from Chan et al. [33].

^‡^Adapted from Distler and Dreher [34].
